# epiACO - a method for identifying epistasis based on ant Colony optimization algorithm

**DOI:** 10.1186/s13040-017-0143-7

**Published:** 2017-07-06

**Authors:** Yingxia Sun, Junliang Shang, Jin-Xing Liu, Shengjun Li, Chun-Hou Zheng

**Affiliations:** 1 School of Information Science and Engineering, Qufu Normal University, Rizhao, 276826 China; 2 Institute of Network Computing, Qufu Normal University, Rizhao, 276826 China; 3 College of Electrical Engineering and Automation, Anhui University, Hefei, 230601 China

**Keywords:** Epistatic interactions, Ant colony optimization, Bayesian network, Mutual information

## Abstract

**Background:**

Identifying epistasis or epistatic interactions, which refer to nonlinear interaction effects of single nucleotide polymorphisms (SNPs), is essential to understand disease susceptibility and to detect genetic architectures underlying complex diseases. Though many works have been done for identifying epistatic interactions, due to their methodological and computational challenges, the algorithmic development is still ongoing.

**Results:**

In this study, a method epiACO is proposed to identify epistatic interactions, which based on ant colony optimization algorithm. Highlights of epiACO are the introduced fitness function *Svalue*, path selection strategies, and a memory based strategy. The *Svalue* leverages the advantages of both mutual information and Bayesian network to effectively and efficiently measure associations between SNP combinations and the phenotype. Two path selection strategies, i.e., probabilistic path selection strategy and stochastic path selection strategy, are provided to adaptively guide ant behaviors of exploration and exploitation. The memory based strategy is designed to retain candidate solutions found in the previous iterations, and compare them to solutions of the current iteration to generate new candidate solutions, yielding a more accurate way for identifying epistasis.

**Conclusions:**

Experiments of epiACO and its comparison with other recent methods epiMODE, TEAM, BOOST, SNPRuler, AntEpiSeeker, AntMiner, MACOED, and IACO are performed on both simulation data sets and a real data set of age-related macular degeneration. Results show that epiACO is promising in identifying epistasis and might be an alternative to existing methods.

## Background

It has been widely accepted that genome-wide association studies (GWAS) play a great role on understanding disease susceptibility and genetic architectures underlying complex diseases, and lots of single nucleotide polymorphisms (SNPs) speculated to associate with diseases have been identified. Nevertheless, most of them seem to be responsible for the causation of Mendelian diseases [1], and they have poor ability in explaining non-Mendelian diseases, i.e., complex diseases, such as cancer, heart disease, cardiopathy, Alzheimer’s disease, hypertension and many others [1, 2]. Recent advances in GWAS confirm that nonlinear interaction effects of SNPs, namely, epistasis or epistatic interactions, could unveil a large portion of unexplained heritability of complex diseases [2]. Therefore, many algorithms have been proposed for identifying epistasis. However, due to their methodological and computational challenges, the algorithmic development is still ongoing [1, 3, 4].

Current epistasis detection methods can be mainly classified into three categories based on their search strategies: exhaustive search [5–7], stochastic search [8–10], and heuristic search [2, 11–15]. Exhaustive search evaluates the associations of all SNP combinations with the phenotype. Wan et al. [5] developed BOOST (BOolean Operation-based Screening and Testing) which has two stages to analyze all pairwise epistatic interactions in genome-wide case-control studies. Zhang et al. [6] proposed TEAM (Tree-based Epistasis Association Mapping) which updates contingency tables by utilizing the structure of a minimum spanning tree to search two-SNP epistatic interactions. Ritchie et al. [7] used MDR (Multifactor Dimensionality Reduction) to identify epistasis, which divides genotypes into low-risk and high-risk groups to reduce search space. As far as we know, MDR is one of the most popular methods in this field. Though these methods show great performance on identifying epistatic interactions in small scale data sets, they have poor ability on large scale data sets due to their exponential time complexities [1, 3]. Performance of stochastic search methods depend on their sampling numbers, and therefore they are not suitable for genome-wide scale data sets [13]. Tang et al. [8] used epiMODE (*epi*static MOdule DEtection) to detect multiple-SNP epistatic interactions by introducing the concept of epistatic module and designing a Gibbs sampling strategy. Jiang et al. [9] proposed epiForest (detection of *epi*static interactions using random *Forest*) which uses random forest to detect epistatic interactions in case-control studies. Zhang et al. [10] developed BEAM (Bayesian Epistasis Association Mapping) to identify epistasis which categorizes SNPs into three non-overlapping groups based on their posterior probabilities. Heuristic search methods generally obtain solutions at substantially reduced time costs, based on their introducing heuristic information and prior knowledge of biological data. For instance, Wan et al. [11] proposed SNPRuler to identify epistatic interactions using predictive rules. Though heuristic search methods sometimes miss global optimal solutions and only obtain several local optimal solutions, they are still promising methods so far.

More recently, many ant colony optimization (ACO) based methods, which belong to the family of heuristic search methods, have been reported for identifying epistatic interactions. Wang et al. [12, 15] proposed AntEpiSeeker and AntEpiSeeker2.0 based on ACO with the two-stage design to detect epistatic interactions. Both of them perform well in their respective experiments though they require large amounts of ants over numerous iterations to obtain acceptable solutions [15]. Shang et al. [2] developed AntMiner to detect epistatic interactions, which is a generalized method of AntEpiSeeker by incorporating heuristic information into ant-decision rule. Although in terms of detection power, it wins most of compared methods, AntMiner needs unaffordable running time to obtain results, which hinders its widely application. Jing et al. [13] proposed MACOED for detecting epistatic interactions, which is a multi-objective ACO supervised heuristic search method, combining both logistical regression and Bayesian network. Though experiments show that MACOED outperforms other compared methods in both detection power and computational feasibility for large data sets, its pheromone updating strategy is not as effective as it claims to be. Our previously proposed method IACO [16] is an alternative to existing ACO based methods, but it is sensitive to SNPs displaying strong marginal effects.

In this paper, we develop a method epiACO to identify epistatic interactions, which based on ACO algorithm. Highlights of epiACO are the introduced fitness function *Svalue*, path selection strategies, and a memory based strategy. The *Svalue* leverages the advantages of both mutual information and Bayesian network to effectively and efficiently measure associations between SNP combinations and the phenotype. Two path selection strategies, i.e., probabilistic path selection strategy and stochastic path selection strategy, are provided to adaptively guide ant behaviors of exploration and exploitation. The memory based strategy is designed to retain candidate solutions found in the previous iterations, and compare them to solutions of the current iteration to generate new candidate solutions, yielding a more accurate way for identifying epistasis. Experiments of epiACO and its comparison with other recent methods epiMODE, TEAM, BOOST, SNPRuler, AntEpiSeeker, AntMiner, MACOED, and IACO are performed on both simulation data sets and an age-related macular degeneration real data set. Results show that epiACO is promising in identifying epistatic interactions and might be an alternative to existing methods. The Matlab version of epiACO running on Microsoft Windows is available online at: https://sourceforge.net/projects/epiaco1/files/epiACO.rar/download.

## Methods

### General ant Colony optimization (ACO)

The general ACO takes inspiration from the foraging behavior of some ant species. Ants walking to and from a food source communicate with each other indirectly by secreting pheromones on the ground, which will gradually evaporate as time passes. The subsequent ants perceive the presence of pheromones and tend to follow the paths with higher pheromone concentration [17]. Such a mechanism forms a positive feedback and eventually most of the ants, if not all, are able to transport food to their nest in the shortest path.

Mathematically, each ant choose its next position through a probability density function (PDF), which is updated by pheromones and heuristic information. The PDF of the general ACO is defined as

1 $$ {P}_k^{ij}(t)=\left\{\begin{array}{c}\hfill \frac{\tau_{ij}{(t)}^{\alpha}{\eta_{ij}}^{\beta}}{\sum_{u\in {U}_k(t)}{\tau}_{iu}{(t)}^{\alpha}{\eta_{iu}}^{\beta}}\kern3em  i\in {U}_k(t)\hfill \\ {}\hfill 0\kern9.5em  otherwise\hfill \end{array}\right. $$

where ${P}_k^{ij}(t)$ is the probability of ant *k* that chooses the next position *j* from the current position *i* at iteration *t*. *τ*_*ij*_(*t*) is the pheromones of the path from position *i* to position *j* at iteration *t.* The heuristic information of the path from position *i* to position *j* is denoted as *η*_*ij*_. *α* and *β* are parameters that control the importances of pheromones and heuristic information respectively. The positions that are not selected so far by ant *k* at iteration *t* are stored in *U*_*k*_(*t*).

In ACO, ants find the optimal solutions through continuous updating pheromones. Pheromones of the path from position *i* to position *j* at iteration *t+*1 are updated according to the formula

2 $$ {\tau}_{ij}\left( t+1\right)=\left(1-\rho \right){\tau}_{ij}(t)+\varDelta {\tau}_{ij}(t) $$

where *ρ* is a evaporation coefficient, and Δ*τ*_*ij*_(*t*) is the increment of pheromones of the path from position *i* to position *j* at iteration *t*, which is defined as

3 $$ \varDelta {\tau}_{ij}(t)=\sum_{k=1}^m\varDelta {\tau}_{ij}^k(t) $$

4 $$ \varDelta {\tau}_{ij}^k(t)=\left\{\begin{array}{c}\hfill \frac{Q}{S_k(t)}\kern1.5em  if\  ant\  k\  via\  t he\  path\  of\  ij\  at\  iteration\  t\hfill \\ {}\hfill 0\kern15.25em  otherwise\hfill \end{array}\right. $$

where *m* is the number of ants. The increment of pheromones of the path from position *i* to position *j* for ant *k* at iteration *t* is denoted as $\Delta {\tau}_{ij}^k(t)$. The path length for ant *k* at iteration *t* is denoted as *S*_*k*_(*t*). *Q* is a user-specified positive constant.

### Fitness function *Svalue*

#### Mutual information

In probability theory and information theory, the mutual information of two random variables is a measure of the mutual dependence between the two variables. More specifically, it quantifies the amount of information obtained about one random variable, through the other random variable. The mutual information is usually used as an association measure in feature selection problems [2, 18, 19]. In epiACO, mutual information is used to measure the associations between SNP combinations and the phenotype, which can be written as

5 $$ MI\left( S; Y\right)= H(S)+ H(Y)- H\left( S, Y\right) $$

where *H*(*S*) is the entropy of *S*, *H*(*Y*) is the entropy of *Y*, *H*(*S,Y*) is the joint entropy of both *S* and *Y*. Here, *S* represents a SNP combination, and *Y* represents the phenotype.

The entropy and the joint entropy are defined as

6 $$ H(S)=-\sum_{j_1=1}^3\cdots \sum_{j_K=1}^3\left( p\left({s}_{j_1},,\cdots, {s}_{j_K}\right)\cdot \log\ p\left({s}_{j_1},,\cdots, {s}_{j_K}\right)\right) $$

7 $$ H(Y)=-\sum_{j=0}^1\left( p\left({y}_j\right)\cdot \log\ p\left({y}_j\right)\right) $$

8 $$ H\left( S, Y\right)=-\sum_{j_1=1}^3\cdots \sum_{j_K=1}^3\sum_{j=0}^1\left( p\left({s}_{j_1},,\cdots, {s}_{j_K},{y}_j\right)\cdot \log\ p\left({s}_{j_1},,\cdots, {s}_{j_K},{y}_j\right)\right) $$

where *s* is the genotype of a SNP, coded as {1, 2, 3} corresponding to homozygous common genotype, heterozygous genotype, and homozygous minor genotype; *y* is the label of a sample, coded as {0, 1} corresponding to control and case; and *p*(⋅) is the PDF.

It is seen that a greater mutual information value means a stronger association between the SNP combination and the phenotype.

#### Bayesian network

The Bayesian network is a probabilistic graphical model that represents a set of random variables and their conditional dependencies via a directed acyclic graph (DAG) [20]. In general, the Bayes theorem can be written as

9 $$ P\left( N| D\right)=\frac{P\left( D| N\right) P(N)}{P(D)} $$

where *P*(*N*|*D*) is the posterior probability of the Bayesian network model *N* given the data *D*, *P*(*D*|*N*) is the class-conditional density, *P*(*D*) is the probability of *D*, and *P*(*N*) is the prior probability of *N*.

In the DAG, the joint probability distribution of *n* nodes, e.g., *x*_1_ , *x*_2_ ,  ⋯  , *x*_*n*_, is defined as

10 $$ p\left({x}_1,{x}_2,,\cdots, {x}_n\right)=\prod_{i=1}^n p\left({x}_i| parent\left({x}_i\right)\right) $$

where *parent*(*x*_*i*_) represents the parent node of *x*_*i*_. In this study, SNPs and the phenotype are denoted as nodes of DAG respectively [21–24]. The directed edges going from SNP nodes to the phenotype node implies that this SNP combination shows correlation with the phenotype [25]. According to Eq. (9), *P*(*D*|*N*) can be computed while all variables in the DAG are discrete values,

11 $$ P\left( D| N\right)=\prod_{i=1}^I\left(\frac{\Gamma \left(\sum_{j=1}^J{\alpha}_{i j}\right)}{\Gamma \left({r}_i+\sum_{j=1}^J{\alpha}_{i j}\right)}\prod_{j=1}^J\frac{\Gamma \left({r}_{i j}+{\alpha}_{i j}\right)}{\Gamma \left({\alpha}_{i j}\right)}\right) $$

where *I* is the combinatorial number of SNP nodes with different values. Specifically, if *l*-SNP nodes link to the phenotype node, the combinatorial number of SNP nodes is 3^*l*^ since one SNP has three genotypes. *J* is the state number of the phenotype node, which is equal to 2. *r*_*i*_ is the number of cases with SNP nodes taking the *i*_*th*_ combination. *r*_*ij*_ is the number of cases with the phenotype node taking the *j*_*th*_ state and its parents taking the *i*_*th*_ combination. *α*_*ij*_ is a parameter that refers to the prior belief about the number of cases while the nodes taking the corresponding *i*_*th*_ combination and *j*_*th*_ state [13]. More often, in order to take an equal likelihood for all possible distributions in each Bayesian network model, *P*(*N*), *P*(*D*) are usually set to constants and *α*_*ij*_ = 1. Then we obtain the following formula,

12 $$ P\left( N| D\right)\propto \prod_{i=1}^I\left(\frac{\left( J-1\right)!}{\left({r}_i+ J-1\right)!}\prod_{j=1}^J{r}_{i j}!\right) $$

On the basis of previous studies [22, 26, 27], K2 score is introduced and defined as

13 $$ \mathrm{K}2\kern0.4em \mathrm{score}=\prod_{i=1}^I\left(\frac{\left( J-1\right)!}{\left({r}_i+ J-1\right)!}\prod_{j=1}^J{r}_{i j}!\right) $$

Besides, logarithmic form of the K2 score can be written as

14 $$ \mathrm{K}2{\ \mathrm{score}}_{\log }=\sum_{i=1}^I\left(\sum_{b=1}^{r_i+1} \log (b)\hbox{-} \sum_{j=1}^J\sum_{d=1}^{r_{i j}} \log (d)\right) $$

It is seen that a lower K2 score implies a greater correlation between the SNP combination and the phenotype.

#### Svalue

In epiACO, the fitness function plays an important role on deciding which SNP combinations are selected as the optimal solutions. Consequently, a novel fitness function *Svalue* is introduced, which combines both mutual information and Bayesian Network. The mutual information can effectively measure the nonlinear relationships between SNP combinations and the phenotype without a complex modeling [28]. The Bayesian network is widely used as a promising measure for measuring dependence of several variables [27]. In order to leverage the advantages of these two measures, *Svalue* is defined as

15 $$ Svalue(A)=\frac{MI}{{\mathrm{K}2\ \mathrm{score}}_{\log }} $$

It is seen that the higher the *Svalue* score, the stronger the association between the SNP combination and the phenotype.

### ACO based method epiACO for identifying epistasis

**Fig. 1:**
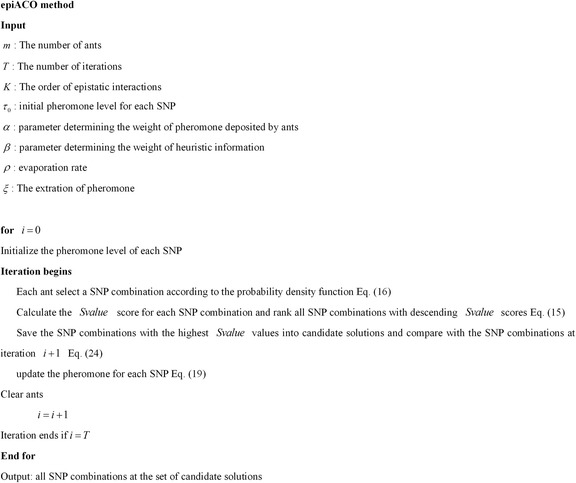
The pseudo code of epiACO

The pseudo code of epiACO is given in Fig. 1, which is mainly consist three strategies, namely, the path selection strategy, the pheromone updating strategy, and the memory based strategy.

#### Path selection strategy

In epiACO, *m* ants are represented by {*m*_1_,  ⋯ , *m*_*k*_,  ⋯ , *m*_*m*_} respectively, each of which is used to evaluate a *K*-SNP combination at each iteration, where *K* is a user-specified order. The probability of ant *k* selecting SNP *i* at iteration *t* is denoted as ${P}_k^i(t)$, which is defined as

16 $$ {P}_k^i(t)=\left\{\begin{array}{c}\hfill R\kern2em  q\le {q}_0\hfill \\ {}\hfill S\kern2em  q>{q}_0\hfill \end{array}\right. $$

In order to control the rate of convergence, avoid falling into local optimal solution, two path selection strategies, that is, probabilistic path selection strategy and stochastic path selection strategy, are provided to adaptively guide ant behaviors of exploration and exploitation. *q*_0_ is a threshold, which defined as the ratio of current iteration number to the total iteration number. *q* is a number that randomly generated from the uniform distribution of [0, 1] while Eq. (16) is employed.

The probabilistic path selection strategy is defined as

17 $$ R=\left\{\begin{array}{c}\hfill \frac{\tau_i{(t)}^{\alpha}{\eta_i}^{\beta}}{\sum_{u\in {U}_k(t)}{\tau}_u{(t)}^{\alpha}{\eta_u}^{\beta}}\kern3em  i\in {U}_k(t)\hfill \\ {}\hfill 0\kern9.5em  otherwise\hfill \end{array}\right. $$

where *τ*_*i*_(*t*) is the amount of pheromones for SNP *i* at iteration *t* and *η*_*i*_ is the heuristic information of SNP *i. U*_*k*_(*t*) is a set of SNPs that are not selected by ant *k* at iteration *t*.

The stochastic path selection strategy is defined as

18 $$ S=\left\{\begin{array}{l}\hfill 1\kern2.25em  i= rand\left({V}_k(t)\right)\ \hfill \\ {}\hfill 0\kern4.25em  otherwise\kern2.75em \hfill \end{array}\right. $$

where all SNPs at iteration *t* is sorted with the descending pheromones, and the latter half is represented as *V*_*k*_(*t*).

This strategy allows epiACO to cover a wider search space while the iteration number is small and to converge on promising regions of the search space while the iteration number turns to large.

#### Pheromone updating strategy

In epiACO, the pheromones of all SNPs are updated according to the following formula

19 $$ {\tau}_i\left( t+1\right)=\left(1-\rho \right){\tau}_i(t)+\varDelta {\tau}_i(t)+\Delta {\tau}_i^{\ast }(t) $$

where Δ*τ*_*i*_(*t*) is the increment of pheromones of SNP *i* at iteration *t*. Besides, additional pheromone increment Δ*τ*_*i*_^∗^(*t*) is adopted to reward the SNPs that belong to the candidate solutions.

The Δ*τ*_*i*_(*t*) and the Δ*τ*_*i*_^∗^(*t*) are respectively defined as

20 $$ \Delta {\tau}_i(t)=\sum_{k=1}^m\Delta {\tau}_i^k(t) $$

21 $$ \varDelta {\tau_i}^k(t)=\left\{\begin{array}{c}\hfill Svalue(A)\kern2em  k\in {M}_i(t)\hfill \\ {}\hfill 0\kern5.25em  otherwise\hfill \end{array}\right. $$

22 $$ \Delta {\tau}_i^{\ast }(t)=\sum_{k=1}^m\Delta {\tau_i^k}^{\ast }(t) $$

23 $$ \varDelta {{\tau_i}^k}^{\ast }(t)=\left\{\begin{array}{c}\hfill \xi Svalue(A)\kern2em  k\in {N}_i(t)\hfill \\ {}\hfill 0\kern5.25em  otherwise\hfill \end{array}\right. $$

where *M*_*i*_(*t*) is the set of ants that select SNP *i* at iteration *t. A* is the SNP combination that has been selected. *Svalue*(*A*) is the *Svalue* of SNP combination *A. N*_*i*_(*t*) is the set of ants that select SNP *i* which belongs to candidate solutions. *ξ* is a specified parameter that used to control the additional increment of pheromones, and the recommended range is [0.2, 0.5].

#### Memory based strategy

In epiACO, SNP combinations that selected by ants at each iteration are evaluated by the fitness function *Svalue*. In order to retain candidate solutions with high *Svalue* scores found in the previous iterations, instead of traditional ACO completely discarding the solutions from previous iterations due to each iteration is independent of each other, a memory based strategy is introduced. First, all SNP combinations selected at iteration *t* are ranked with the descending *Svalue* scores. Second, an inflection point of these descending *Svalue* scores is computed using the formula

24 $$ f= arc\underset{g=3}{\overset{m}{ \max }}\left(\left( Svalue\left({A}_g\right)- Svalue\left({A}_{g-1}\right)\right)-\left( Svalue\left({A}_{g-1}\right)- Svalue\left({A}_{g-2}\right)\right)\right) $$

where *A*_*g*_ is the SNP combination and *Svalue*(*A*_*g*_) is the *Svalue* score of the SNP combination *A*_*g*_. Third, the SNP combinations with top *f Svalue* scores are viewed as the candidate solutions. Fourth, candidate solutions of previous iterations are compared to solutions of the current iteration to generate new candidate solutions, which will continue to be used in the next iteration. The merit of this strategy is that good solutions generated in any of the iterations will not be lost, yielding a more accurate way for epistasis searching.

## Results and discussion

### Evaluation measures

Detection power is one of the generally accepted and widely used evaluation measure in the field of identifying epistasis [2, 8, 10, 11, 29–31]. In this study, we directly use the detection power proposed by previous studies [2, 8, 10, 11, 29–31], which is defined as the proportion of data sets in which the epistasis models are perfectly identified with no false positives. That is,

25 $$ \mathrm{Power}=\frac{R}{G} $$

where *R* is the number of data sets that epistasis models in them are successfully detected, *G* is the number of all experimental data sets.

Computational complexity is also analyzed. We measure running time in the same computational environment to assess realistic applicability of compared methods (Intel G640 2.80GHz CPUs, 32GB RAM, Microsoft Windows, MATLAB).

### Simulation and real data sets

We exemplify 5 commonly used benchmark models of 2-SNP epistatic interactions for the study [8, 10, 31–34]. Details of the models are given in Table 1. Specifically, Model 1 is the model that displays both marginal effects and interaction effect (ME model), the penetrance of Model 1 increases only when both SNPs have at least one minor allele [8, 10]; Model 2 is also the ME model that display both marginal effects and interaction effect, the additional minor allele at each SNP of which does not further increase the penetrance [10]; Model 3 is randomly chosen from references [33, 34] that show no marginal effects but interaction effect (NME model); Model 4 is directly cited from the reference [33], which is also a NME model; Model 5 is a ZZ NME model [32].

**Table 1:** Details of 5 commonly used benchmark models of 2-SNP epistatic interactions

Models		MAF(*a*)	MAF(*b*)	Prevalence	Penetrance function			
					Genotypes (SNP *A*)	Genotypes (SNP *B*)		
						*BB*	*Bb*	*bb*
ME Models	Model 1	0.300	0.200	0.100	*AA*	0.087	0.087	0.087
					*Aa*	0.087	0.146	0.190
					*aa*	0.087	0.190	0.247
	Model 2	0.400	0.400	0.050	*AA*	0.042	0.042	0.042
					*Aa*	0.240	0.270	0.420
					*aa*	0.240	0.440	0.090
NME Models	Model 3	0.500	0.500	0.300	*AA*	0.470	0.230	0.270
					*Aa*	0.240	0.270	0.420
					*aa*	0.240	0.440	0.090
	Model 4	0.400	0.400	0.171	*AA*	0.068	0.299	0.017
					*Aa*	0.289	0.044	0.285
					*aa*	0.048	0.262	0.174
	Model 5	0.500	0.500	0.010	*AA*	0.000	0.000	0.100
					*Aa*	0.000	0.050	0.000
					*aa*	0.100	0.000	0.000

Prevalence is the proportion of samples that occur a disease. Penetrance is the probability of the occurrence of a disease given a particular genotype.MAF(*a*) is the minor allele frequency of *a*. *AA*, *Aa*, and *aa* are homozygous common genotype, heterozygous genotype, and homozygous minor genotype

For each model, 100 data sets are simulated by epiSIM [35], each containing 2000 cases and 2000 controls. In the first 50 data sets, 100 SNPs are genotyped, while in other 50 data sets the number of genotyped SNPs is increased to 1000. For each data set, random SNPs are set with their minor allele frequencies chosen from [0.05, 0.5] uniformly.

A real data set of age-related macular degeneration (AMD) is also used for testing epiACO. AMD, refers to pathological changes in the central area of the retina, is the most important cause of irreversible visual loss in elderly populations, and is considered as a complex disease having multiple epistatic interactions [8]. The AMD data set contains 103,611 SNPs genotyped by 96 cases and 50 controls, which has been widely used as a benchmark data set [2, 8, 9, 12, 13, 15, 16, 29, 36, 37].

### Experiments on simulation data sets

In the study, performance of epiACO is analyzed by comparison with 8 typical 2-SNP epistasis detection methods, i.e., epiMODE, TEAM, BOOST, SNPRuler, AntEpiSeeker, AntMiner, MACOED, and IACO. Among them, BOOST and TEAM are exhaustive search methods, epiMODE is a stochastic search method, others are heuristic search methods. In particular, AntEpiSeeker, AntMiner, MACOED, and IACO are all ACO based methods, which will be discussed in more detail than others since they belong to the same family, as well as epiACO.

The first experiment is performed on 100-SNP data sets. The parameters of each compared method are generally set as default. Only a few are modified according to suggestions in their respective user manual to balance result accuracy and computational cost. For epiMODE, the iteration number is set to 100. For TEAM, the permutation number is set to 100. For BOOST, the iteration threshold is set to 10. For a fair comparison, parameter settings of the ACO based methods are same. Specifically, the iteration number *m* and the ant number *T* are set to 25 and 200 respectively. The initial pheromone *τ*_0_, the heuristic information *η*, and the parameters *α* and *β* are all set to 1. The evaporation coefficient *ρ* is set to 0.2. The constant *ξ* is set to 0.3. For obtaining accurate results, each method runs 20 times with different random seeds on each data set of each model, which can ensure that the method has not been biased by its initial starting conditions. The average detection power and the average running time with their respective standard deviations on 100-SNP data sets are recorded in Table 2. It is seen that epiACO is comparable and sometimes superior to compared methods, especially ACO based methods.

**Table 2:** The average detection power and the average running time with their respective standard deviations of compared methods on 100-SNP data sets

Evaluation measures	Models		epiMODE	TEAM	BOOST	SNPRuler	AntEpiSeeker	AntMiner	MACOED	IACO	epiACO
Detection power (%)	ME Models	Model 1	45.42 ± 3.58	89.75 ± 1.22	0.00 ± 0.00	0.00 ± 0.00	81.95 ± 2.57	89.51 ± 1.67	76.45 ± 5.10	100.00 ± 0.0	100.00 ± 0.0
		Model 2	0.00 ± 0.00	14.03 ± 0.15	0.00 ± 0.00	0.00 ± 0.00	33.57 ± 7.28	86.75 ± 4.36	84.52 ± 4.90	94.37 ± 1.84	97.85 ± 1.34
	NME Modes	Model 3	0.00 ± 0.00	100.00 ± 0.0	100.00 ± 0	100.00 ± 0.0	26.83 ± 6.25	85.47 ± 5.00	74.67 ± 4.90	26.83 ± 6.08	75.25 ± 5.83
		Model 4	0.00 ± .000	79.77 ± 1.76	100.00 ± 0	100.00 ± 0.0	42.33 ± 7.87	100.00 ± 0.0	82.13 ± 3.74	30.14 ± 7.21	81.87 ± 6.33
		Model 5	0.00 ± .000	0.00 ± 0.00	100.00 ± 0	98.52 ± 1.48	76.45 ± 4.12	100.00 ± 0.0	76.33 ± 3.46	44.67 ± 11.4	75.62 ± 7.75
Running time (second)	ME Models	Model 1	98.22 ± 0.81	35.13 ± 0.26	0.33 ± 0.06	0.93 ± 0.02	28.73 ± 0.50	1100.50 ± 26.08	52.56 ± 0.57	25.03 ± 0.45	23.48 ± 0.44
		Model 2	95.70 ± 0.75	34.78 ± 0.26	0.28 ± 0.04	0.96 ± 0.03	29.08 ± 0.54	1101.60 ± 65.09	53.64 ± 0.77	28.22 ± 0.56	25.07 ± 0.49
	NME Models	Model 3	97.36 ± 0.64	35.02 ± 0.27	0.27 ± 0.03	0.97 ± 0.02	28.74 ± 0.35	1002.90 ± 52.95	59.92 ± 0.57	26.78 ± 0.77	25.77 ± 0.37
		Model 4	99.21 ± 0.83	34.90 ± 0.26	0.29 ± 0.05	0.98 ± 0.02	28.14 ± 0.40	1017.00 ± 36.01	59.68 ± 0.70	26.21 ± 0.44	18.29 ± 0.37
		Model 5	95.89 ± 0.70	35.22 ± 0.26	0.28 ± 0.04	0.97 ± 0.02	28.77 ± 0.53	1102.30 ± 27.59	52.76 ± 0.66	26.14 ± 0.37	25.19 ± 0.47

For ACO based compared methods, epiACO outperforms AntEpiSeeker and IACO in terms of detection power on all models while they have the similar running time. epiACO has higher detection power than those of AntMiner and MACOED on ME models, but lower detection power than those of them on NME models. Since ME models are the primary models of epistatic interactions in real genetic architectures underlying complex diseases and NME models are special cases, epiACO is more suitable for real applications than other two methods. AntMiner has higher detection power than that of epiACO on NME models due to heuristic information of SNPs being incorporated into its ant-decision rules, which on one hand improves detection power, and on the other hand significantly increases running time, hindering its widely application on large scale data sets, like those for GWAS. For MACOED, it is in fact the stochastic search method since its pheromone updating strategy is only used for more frequently detecting the epistatic interactions that have been detected in the previous iterations, rather than to identify better epistatic interactions. As it is known to all, stochastic search methods are good at identifying NME models, and hence detection power of MACOED on NME models are higher than that of epiACO.

For other compared methods, their results are consistent with and complementary to previous reported results [31]. Both BOOST and SNPRuler have perfect detection power on NME models but detect nothing on ME models since they only focus on identifying NME models. TEAM performs well on some models and worse on others, implying that it is model sensitive. epiMODE detects nothing in four models and only has moderate detection power on Model 1. On the contrary, epiACO performs well on all models, either NME models or ME models, shows that epiACO is model-free and has high stability and reliability.

The second experiment is performed on 1000-SNP data sets. The purpose of this experiment is to demonstrate that epiACO has a place for larger data sets and performs better than compared methods. AntEpiSeeker, MACOED, IACO and epiACO are compared on these data sets with the parameters of the iteration number being 100 and the ant number being 500. Other parameters are the same as those of the first experiment. TEAM, epiMODE, AntMiner and SNPRuler are not considered here due to their unaffordable computational cost or memory on high-dimensional data sets [31]. Since BOOST is an exhaustive search method only focusing on NME models, its results on 1000-SNP data sets can be inferred that it detects nothing on ME models and has perfect detection power on NME models. Each compared method also runs 20 times with different random seeds on each data set of each model to ensure that the method has not been biased by its initial starting conditions. The average detection power and the average running time with their respective standard deviations on 1000-SNP data sets are recorded in Table 3. It is seen that epiACO indeed performs better on larger data sets than compared methods. Specifically, it is the fastest one among the methods; though detection power does not reach a perfect level due to the small iteration number and the ant number, epiACO is still the winner.

**Table 3:** The average detection power and the average running time with their respective standard deviations of compared methods on 1000-SNP data sets

Evaluation measures	Models		AntEpiSeeker	MACOED	IACO	epiACO
Detection power (%)	ME Models	Model 1	36.10 ± 3.86	16.33 ± 5.16	100.00 ± 0.00	100.00 ± 0.00
		Model 2	0.00 ± .0.00	18.14 ± 4.75	17.62 ± 4.67	35.33 ± 3.26
	NME Modes	Model 3	0.00 ± .0.00	9.80 ± 2.74	7.00 ± 2.93	10.30 ± 2.69
		Model 4	0.00 ± .0.00	6.10 ± 2.94	4.90 ± 2.19	9.90 ± 2.83
		Model 5	0.00 ± .0.00	9.60 ± 3.34	5.70 ± 2.77	11.23 ± 3.57
Running time (second)	ME Models	Model 1	357.11 ± 13.08	323.81 ± 18.47	301.27 ± 13.43	231.38 ± 12.65
		Model 2	305.40 ± 16.33	334.01 ± 17.74	270.31 ± 18.91	229.33 ± 15.91
	NME Models	Model 3	281.08 ± 17.23	337.49 ± 16.72	253.00 ± 18.82	253.41 ± 12.57
		Model 4	262.75 ± 14.26	345.21 ± 19.41	277.04 ± 14.37	241.41 ± 10.02
		Model 5	305.99 ± 14.88	354.87 ± 13.48	314.40 ± 12.35	267.49 ± 11.63

**Fig. 2:**
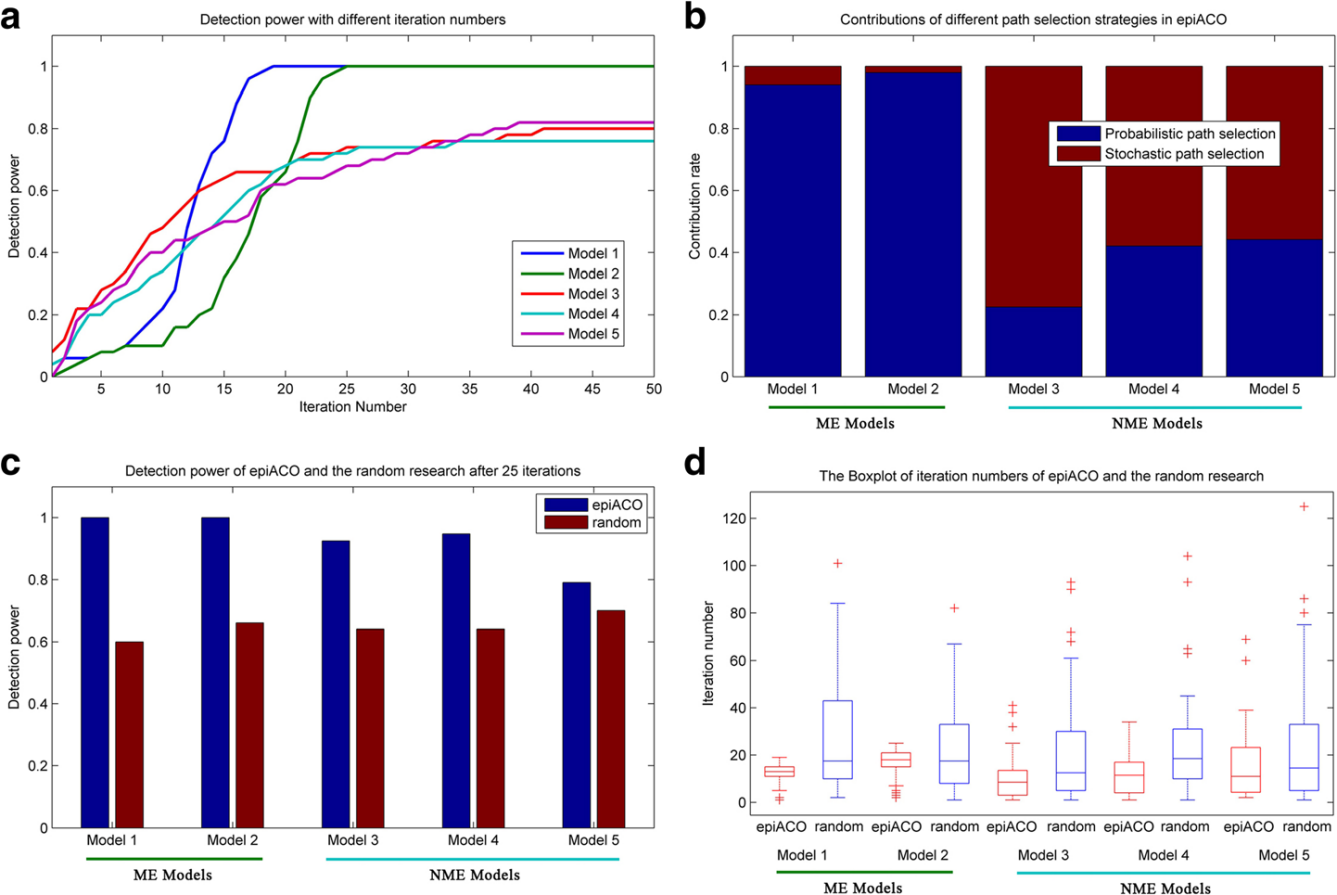
The convergence and contribution of epiACO with the parameter settings being the same as those of the first experiment

The third experiment is performed on 100-SNP data sets to test the convergence and contribution of epiACO with the parameter settings being the same as those of the first experiment, and results of which are shown in Fig. 2. From Fig. 2a, it is seen that detection power of all models increase as the iteration numbers increase, and gradually tend to be stable while the iteration numbers come to 25, which might be the evidence that epiACO has converged after 25 iterations in the first experiment. The Fig. 2b shows the contributions of different path selection strategies in epiACO. It is clear that the probabilistic path selection strategy plays a decisive role on ME models while the stochastic selection strategy plays a decisive role on NME models. Therefore, they supplement each other and both of them are indispensable for epiACO. In order to test the contribution of the ant system, both epiACO and a simple random search are run on all models with 200 ants. Fig. 2c records the detection power of them after 25 iterations, and Fig. 2d is a box plot of iteration numbers of them while obtaining the global optimal solutions. Results in these two subfigures show that the detection power of epiACO after 25 iterations is higher than that of the random search on all models, which implies that the introduced ACO system improves the performance of the algorithm.

### Application to real data set

Potential of the epiACO can also be verified by analyzing a real AMD data set with different parameter settings (*m*, *T*, *ρ*) being (10,000, 500, 0.2) and (20,000, 250, 0.2). The captured 2-SNP epistatic interactions that might be associated with the AMD are reported in Table 4 with ascending Chi-square *p*-values.

**Table 4:** Top 10 captured epistatic interactions associated with AMD. *CFH*: complement factor H. *N/A*: no gene is available. *MED27*: mediator complex subunit 27. *KDM4C*: lysine (K)-specific demethylase 4C. *NCALD:* neurocalcin delta. *ISCA1*: iron-sulfur cluster assembly 1. *NEDD9*: neural precursor cell expressed developmentally down-regulated 9

SNP 1			SNP 2			*P*-value
Name	Gene	Chromosome	Name	Gene	Chromosome	
rs380390	*CFH*	1	rs1363688	*N/A*	5	4.5728 e-09
rs380390	*CFH*	1	rs2224762	*KDM4C*	9	3.2929 e-08
rs380390	*CFH*	1	rs1374431	*N/A*	2	3.9086 e-08
rs1740752	*N/A*	10	rs1368863	*N/A*	11	9.8438 e-08
rs380390	*CFH*	1	rs223607	*N/A*	6	1.9797 e-07
rs1329428	*CFH*	1	rs9328536	*MED27*	9	2.1498 e-07
rs1740752	*N/A*	10	rs943008	*NEDD9*	6	3.8365 e-07
rs380390	*CFH*	1	rs718263	*NCALD*	8	5.1901 e-07
rs380390	*CFH*	1	rs2402053	*N/A*	7	1.1131 e-05
rs380390	*CFH*	1	rs10512174	*ISCA1*	9	3.9083 e-05

It is seen that most reported epistatic interactions contain either rs380390 or rs1329428, which is due to these two SNPs having strongest main effects among all SNPs, leading to their combinations with other SNPs usually displaying strong interaction effects, and hence being identified. SNPs rs380390 and rs1329428 reside in the *CFH* gene, mutations in this gene have been associated with hemolytic-uremic syndrome (HUS) and chronic hypocomplementemic nephropathy. These two SNPs are believed to be significantly associated with AMD [8]. There are only two detected epistatic interactions in the table not containing either rs380390 or rs1329428, that is, (rs1740752, rs1368863) and (rs1740752, rs943008), which are the first time being identified that might be associated with the AMD. Both SNPs of the former locate in the noncoding regions, might lead to AMD through regulating gene expression levels. Obviously this is a bold speculation, needs further studies in depth with the use of more case-control samples, and also the biological experiments, though they are beyond the scope of this study. SNP rs1740752 appears in these two epistatic interactions, but its main effect is moderate, implying not only that it influences the phenotype mainly through its interaction effects with other SNPs, but also that epiACO is promising in identifying epistatic interactions displaying no marginal effect but interaction effects. SNP rs2224762 resides in the intron of *KDM4C* gene, and chromosomal aberrations and changes in expression of this gene may be found in tumor cells. SNP rs943008 resides in the intron of *NEDD9* gene, altered expression of which is strongly associated with cancer. The *NEDD9* overexpression is documented to occur and in some cases linked the process of tumorigenesis of many different malignances. Besides, *NEDD9* has been studies for a possible association with late onset Alzheimer’s disease, and may be important for recovery from stroke. Therefore, mutations in *NEDD9* gene might involve in AMD. We hope that, from these results, some clues could be provided for the exploration of causative factors of AMD.

## Conclusions

It has been widely accepted that complex diseases are mainly caused by epistatic interactions. In the study, a method epiACO based ACO algorithm is presented for detecting epistatic interactions. Highlights of epiACO are the introduced fitness function *Svalue*, path selection strategies, and a memory based strategy. The *Svalue* leverages the advantages of both mutual information and Bayesian network to effectively and efficiently measure associations between SNP combinations and the phenotype. Two path selection strategies, i.e., probabilistic path selection strategy and stochastic path selection strategy, are provided to adaptively guide ant behaviors of exploration and exploitation. The threshold of *q*_0_ allows epiACO to cover a wider search space while the iteration number is small and to converge on promising regions of the search space while the iteration number turns to large, resulting in high detection power not only in ME models but also in NME models. The memory based strategy is designed to retain candidate solutions found in the previous iterations, and compare them to solutions of the current iteration to generate new candidate solutions, yielding a more accurate way for identifying epistasis. Experiments of epiACO and its comparison with other recent methods epiMODE, TEAM, BOOST, SNPRuler, AntEpiSeeker, AntMiner, MACOED, and IACO are performed on both simulation data sets and a real data set of age-related macular degeneration. Results show that epiACO is promising in identifying epistasis and might be an alternative to existing methods.

The aim of this study is to develop an epistasis detection method based on ACO algorithm, which is comparable and sometimes superior to existing methods, especially ACO based methods for identifying epistasis. This does not mean that methods based on other principles are not suitable for identifying epistasis, and their performance are worse than epiACO. We believe that each method has its own merits and limitations, and the more the methods based on different principles being proposed, the faster this problem being solved. For instance, the common standard conjugate gradient algorithm [38] and the genetic algorithm [39] are important optimization algorithms, and can undoubtedly deal with this problem.

Although the results demonstrate that epiACO performs well on both simulation data sets and a real AMD data set, several limitations remain. First, although detection power is the generally accepted and widely used evaluation measure for detecting epistatic interactions [2, 8, 10, 11, 29–31], and we directly use it in this study, other evaluation measures, for example, sensitivity, specificity, accuracy, balanced accuracy, and so on, should be used to carry out a broader performance analysis. Second, several important parameters of epiACO, including the number of ants, the number of iterations and the evaporation coefficient, should be discussed in detail and give their recommended settings respectively. Though how to set parameters appropriately is a great challenge for the family of swarm intelligence algorithms, like ACO algorithm. These limitations inspire us to continue working in the future.

## Abbreviations

ACO: Ant colony optimization
AMD: Age-related macular degeneration
BEAM: Bayesian epistasis association mapping
BOOST: Boolean operation-based screening and testing
DAG: Directed acyclic graph
epiForest: Detection of epistatic interactions using random forest
epiMODE: Epistatic module detection
GWAS: Genome-wide association studies
MDR: Multifactor-dimensionality reduction
ME models: Models that display both marginal effects and interaction effect
NME models: Models that shows only interaction effects
PDF: Probability density function
SNP: Single-nucleotide polymorphism
TEAM: Tree-based epistasis association mapping
